# Diseases of the Tonsils

**Published:** 1894-03-10

**Authors:** 


### DISEASES OF THE TONSILS.

### The Larynx.

*Intubation.*—A report of 70 cases by Dr. R. T. Stanton[4], giving a mortality of 46, or 34·3 per cent. of recovery, is especially worthy of note, as all but two of the cases were tenement house patients, and therefore subject to many disadvantages in the way of nursing and general attendance. Of the 46 deaths, 14 were caused by extension of membranes, five by sepsis, four by asphyxia and pneumonia, three by uræmic convulsions, two by exhaustion, and two each by pulmonary œdema, nephritis, and bronchitis. He states that it is a rule with him never to operate unless in the presence of another medical man, unless the symptoms are too urgent to wait. He has never refused to operate on any patient as being too far gone, but in five or six cases in which he had been called in by the attending physicians to operate he has not done so, as the symptoms were not sufficiently urgent. These statements show us that Dr. Stanton has not by any means picked his cases, and his percentage of recoveries are all the more encouraging.

A contribution to this subject comes from Dr. G. M. Lefferts[5], who, having three years ago discussed the merits of intubation of the larynx in acute and chronic laryngeal stenosis[6], turns now to the other conditions in the adult in which intubation may afford relief. In bilateral abductor paralysis in chronic cases no cure can result of course, but in recent cases dependent upon paralysis of constitutional origin, *e.g.*, syphilis, it is useful. For permanent use, such as would be necessary in chronic cases, tracheotomy possesses advantages over intubation.

But in cases of laryngeal dyspnœa, dependent upon abductive immobility of the vocal cords, due to anchylosis of the arytenoid articulations from either constitutional causes leading to laryngeal affections, or non-suppurative adhesive arthritis due to local inflammatory changes, perhaps to mechanical fraction of the joints from various causes. In such instances, well-directed pressure by means of a series of graded and even specially shaped intubation tubes will, it is believed, exert a favourable influence in promoting the absorption of the effused material, or by forcibly breaking up cicatricial attachments, has freed articular movement.

He refers also to the use of the intubation tube as an intralaryngeal splint in cases of fracture and displacement of the laryngeal cartilages, due to injury; in acute dyspnœa, du e to œdematous infiltration or to the development of hæmatomata.

The tubercular larynx does not commend itself, he says, for many reasons as a proper site for the sojourn of an intubation tube, although a few cases of relief of dyspnœa dependent on chronic œdema in tubercular laryngitis have been reported, and in perichondritis, and for bilateral abductor paresis from joint fixation.

In acute inflammatory affections of the larynx and their results, intubation will play no unimportant *rôle* in future, *e.g.*, in acute subglottic œdema, either in the child—or as rarely occurs—in the adult.

In spasm of the glottis where tracheotomy would be demanded, Dr. Leffert's would substitute intubation.

Progressive intubation has been used successfully in cases with partial adhesion of the vocal cords. Reference is made to Lichwitz fenestrated intubation tubes for the removal of laryngeal growths. Again, when dyspnœa is dependent upon a stenosis caused by excessive granulation tissue springing from the upper borders of the laryngotomy or tracheotomy wound and filling the lower larynx or upper trachea, or when inflammatory swelling or hyperplastic hypertrophy of laryngeal mucous membrane below the glottis and above the limits of the wound into the air passage exist, intubation will be and has been of service.

Eight cases of syphilitic stenosis of the larynx caused by web formation operated on by the method of combined tubage and the knife, are reported by Dr. J. Mount Bleyer,[7] of New York. He has arrived at the following conclusions: (1) The destruction of the cicatricial web by means of the knife is preferable in every way to the older operation of simple dilatation. He uses Lennox Browne's cutting dilator, and finishes the operation by introducing a hard rubber intubation tube. The tubes are taken out, cleaned, and replaced every three days for a fortnight. Astringent solutions should be used in spray form after treatment, and iodide of potassium may be given internally. (2) It is a more radical procedure, and the obstructing tissue is destroyed quickly, instead of being pushed aside and thus allowed to absorb. (3) The operation saves time, a cure being effected with less chance of a recurrence of the difficulty, without increasing the risks of operation, than by means of simple dilatation.

The statistics collected by the German Pædriatric Society, and published by H. V. Ranke,[8] compare the results of tracheotomy and intubation. They tend to show that during the first two years of life intubation gives a much larger percentage of recoveries than tracheotomy, and that the average results at all ages show that intubation compares favourably with tracheotomy,

Dr. Mount Bleyer[9] has published a case showing how a lost intubation tube was positively located by means of an electrical searcher. The searcher consisted of two insulated needles in close apposition, so combined in the form of a "needle probe" that they can together be made to puncture the skin or be introduced into various cavities. Any piece of metal against which it may impinge will, by completing the circuit of a battery in connection with the proximal ends of the needles, set a bell a ringing, and so give notice of the presence of the foreign metallic body. The device proved most useful in Dr. Mount Bleyer's case of intubation tube in the upper part of the trachea.

*Laryngeal Tuberculosis.*—A clinical lecture by Dr. Felix Semon[10] is an authoritative *résumé* of our present knowledge of laryngeal affections in phthisis, which the author states is proved to occur in not less than 20 per cent. of cases of tubercular disease of the lungs. Some important questions are answered by Dr. Semon. Does the laryngeal tuberculosis begin on the surface and penetrate into the lower tissues, or is the order of events just the opposite? He replies that in all probability there is something in both views, but the greater likelihood appears to be in favour of the view that the disease, in a majority of cases, begins on the surface, and from there gradually penetrates into the deeper tissues. He endorses the now generally recognised view, that pharyngeal and laryngeal anæmia is one of the most valuable premonitions of pulmonary tuberculosis. The laryngeal complications may be said to take two directions: (*a*) the specifically tubercular complications, (*b*) the accidental, *e.g.*, chronic laryngitis, and paresis or paralysis of a vocal cord, the latter due either to implication of one of the recurrent laryngeal nerves in pleuritic thickening round the apex of an affected lung (usually the right one is affected), or to that peculiar waxy degeneration of the laryngeal muscles described by Erb.

The diagnosis must be made from simple chronic laryngitis, syphilis, and malignant disease. Chronic laryngitis limited to one vocal cord only is very suspicious of grave organic disease. Syphilis is often difficult to distinguish, but it must be remembered that syphilis and tubercular disease may co-exist in the larynx. A good instance of this was pictured by Dr. Watson Williams[11] in an article on "The Combination of Syphilis and Tuberculosis, Especially in Regard to Laryngeal Affections." So also may malignant disease be associated with tubercular disease of the larynx.

*Voice Culture.*—Dr. H. H. Curtis[12] has for many years been investigating the relation of impairment of the vocal organs resulting from improper methods of voice production. His more recent conclusions are therefore worthy of attention. After briefly reviewing the types of respiration during vocalisation as classified in 1842 by Beau and Maissait, viz., the superior costal, inferior costal, and abdominal, and referring to the advocacy of different schools of these several types, he proceeds to emphasise the importance of voice production with the vocal cords rendered tense in their whole length by means of the cricothyroid muscle. This is brought about when the vocal cords have been properly "poised"—the larynx fixed by filling the upper part of the chest and lowering the chin. "Joal made the assertion, in an article published in 1890, that great artistes, especially women, used the superior costal method. Melchissedec offered himself as an example of this superior costal respiration, saying it had enabled him to sing constantly for twenty-five years."

He strongly condemns the teaching of those who require their pupils to keep their palates up (thereby cutting off the natural resonation, the nasopharynx and nasal passages), to sing in the back of their heads, and strike the glottis. The vocal cords will be seen by the laryngoscope to bulge towards the middle line, and in the initial attack, being in apposition, differing entirely from the condition when superior costal fixation is taught, for then the vocal cords vibrate in their whole length, and even in the initial attack being widely apart. The upper ribs should remain fixed, and the breathing be entirely inferior costal and diaphragmatic. As a consequence of the practical inculcation of the proper method of voice production alone, Dr. Curtis has been enabled to cure conditions of the larynx which made singing impossible, and he has largely dispensed with intralaryngeal medication, and even with rest in such cases.

[4] Med. Rec., Nov. 25, 1893. [5] New York Med. Jour., Dec. 9, 1893. [6] Med. Rec., Oct. 4, 1890. [7] Jour. of Amer. Med. Sc., Nov, 25, 1893. [8] Munich Med. Work, No. 44, 1893. [9] Med. Rec.. Dec. 16, 1893. [10] Clin. Journ., Jan. 3, 1894. [11] Brist. Med. Chi. Journ., Sept., 1893. [12] New York Med. Journ., Jan. 20, 1894.

